# Cancer stem cell induction from mouse embryonic stem cells

**DOI:** 10.3892/ol.2019.10614

**Published:** 2019-07-15

**Authors:** Akimasa Seno, Chikae Murakami, Bishoy El-Aarag, Yoshiaki Iwasaki, Toshiaki Ohara, Masaharu Seno

**Affiliations:** 1 Laboratory of Nano-Biotechnology, Graduate School of Interdisciplinary Science and Engineering in Health Systems, Okayama University, Okayama 700-8530, Japan; 2 Okayama University Research Laboratory for Stem Cell Engineering in Detroit, Integrative Biosciences Center, Wayne State University, Detroit, MI 48202, USA; 3 Department of Medical Bioengineering, Graduate School of Natural Science and Technology, Okayama University, Okayama 700-8530, Japan; 4 Biochemistry Division, Chemistry Department, Faculty of Science, Menoufia University, Shebin El-Kom 32511, Egypt; 5 Health Service Center, Okayama University, Okayama 700-8530, Japan; 6 Department of Pathology and Experimental Medicine, Graduate School of Medicine, Dentistry and Pharmaceutical Sciences, Okayama University, Okayama 700-8558, Japan

**Keywords:** cancer stem cell, mouse embryonic stem cells, conditioned medium

## Abstract

Although cancers are often removed by surgery and treated by chemotherapy and/or radiation therapies, they often reoccur following treatment due to the presence of resistant residual cells such as cancer stem cells (CSCs). CSCs are characterized by their self-renewal, pluripotency, and tumorigenicity properties, and are promising therapeutic targets for the complete therapy of cancers; however, the number of CSCs in cancer tissue is typically too small to investigate fully. We have previously reported that CSCs could be established from induced pluripotent stem cells (iPSCs) using a conditioned medium during cancer cell culture. In the present study, mouse embryonic stem cells (mESCs) were observed to be converted to CSCs (mES-CSCs). This demonstrated that CSC induction does not exclusively occur following gene editing in somatic cells, and that conditioned medium from cancer cells may contain factors that can induce CSCs. Therefore, not only iPSCs but also mESCs, were demonstrated to be able to produce CSCs as one of the potentials of pluripotency of stem cells, suggesting that the conversion to CSCs is not specific to iPSCs. The resultant mES-CSCs would be also useful to generate tissue specific cancers and these naturally occurring cancers can contribute to drug screenings, but also undergo further investigation in order to reveal cancer mechanisms.

## Introduction

Cancer plagues human beings since ancient era and there is still no complete therapy until today. This plague is usually removed from the patients by surgery or sometimes only by radiation or drugs, although, these treatments not always succeed. There are many reasons for these failures, one of them was thought to be the small population of cells that can initiate cancer development and consequently recurrence of cancer. Decades ago, leukemic stem cell was obtained from mice injected with human acute myeloid leukemia cells (1). This finding concreted the existence of cancer stem cells (CSCs) and researchers started to research on them. CSCs have normal stem cell properties like self-renew and multipotency as well as they provide their progenies as cancer cells (2). They can make not only cancer tissue but also cancer microenvironment (3) and contribute to cancer proliferation, invasion, and metastasis. Moreover, due to the resistance to chemo- and radiation-therapy, cancer can recur from survived residual CSCs even after treatment. Therefore, the complete cure of cancer is often difficult, so that the development of effective therapy to treat CSCs should eagerly be expected. For the development of the effective therapy, enough number of CSCs should be required for the characterization. However, it is difficult to obtain sufficient quantities of CSCs for analysis from clinical specimens since only small population of CSCs is present in a cancer tissue.

We have previously demonstrated that CSCs can be artificially induced by culturing mouse induced pluripotent stem cells (iPSCs) in the presence of culture supernatant of cancer cell lines (3–9). In the current study, we induced CSCs from mouse embryonic stem cells (mESCs). They form colony on coated dish, spheroids in suspension culture, and tube structure with appropriate growth factors after induction. They also induce tumor in C57BL/6 mice and keep expressing stem cell markers during induction. Taken together, mESCs can be converted into CSCs and would be a touchstone as CSCs can be derived from normal stem cells.

## Materials and methods

### 

#### Cell culture

Mouse ESCs [B6J-23^URT^ cells (RBRC-AES0143) and B6G-2 cells (RBRC-AES0003)] were purchased from Riken Cell Bank (Tokyo, Japan) and maintained in DMEM (Sigma-Aldrich; Merck KGaA, Darmstadt, Germany) containing 15% FBS (Nichirei, Tokyo, Japan), 0.1 mM NEAA (Invitrogen; Thermo Fisher Scientific, Inc., Waltham, MA, USA), 2 mM L-Glutamine (Sigma-Aldrich; Merck KGaA), 0.1 mM 2-mercaptoethanol (Wako, Osaka, Japan), 1×10^6^ Unit/ml LIF (Wako), 50 U/ml penicillin and 50 µg/ml streptomycin (Wako) on feeder layers of mitomycin-C-treated mouse embryonic fibroblast (MEF) cells (RCHEFC003; Reprocell, Kanagawa, Japan). B6G-2 cells are derived from a transgenic mouse, of which cells are introduced GFP gene into the genome so that GFP should constitutively express (10). Mouse Lewis lung carcinoma (LLC) cells (11,12) (JCRB1348; JCRB cell Bank, Osaka, Japan) and mouse melanoma B16 cells (JCRB0202; JCRB cell Bank) (13–15) were purchased from ATCC (Manassas, VA, USA) and maintained in DMEM (Sigma-Aldrich; Merck KGaA) containing 10% FBS. Mouse melanoma B16 cells were maintained in RPMI1640 (Sigma-Aldrich; Merck KGaA) containing 10% FBS. The cells were incubated at 37°C under the atmosphere of 5% CO_2_.

To prepare the conditioned medium (CM), cell culture supernatant of the different mouse cancer cell lines was collected from confluent dishes and filtered through 0.45 µm filter (EMD Millipore, Billerica, MA, USA). For the CSCs conversion, mESCs (without MEF feeder cells) were maintained in the medium equally mixed from the CM and the fresh medium of mESCs without LIF. The medium was changed every day. Mouse ESCs without the CM treatment were used as controls. Namely, mouse ESCs were cultured in mESCs medium without LIF or equal mixture of mESCs medium and 5% FBS/DMEM without LIF. Mouse ESCs were passaged when they reached 80% confluent. B6J-23^URT^ cells cultured with the CM of LLC cells and B16 cells are termed B6J-LLCcm and B6J-B16cm, respectively. B6G-2 cells cultured with the CM of LLC cells were termed B6G-LLCcm.

For primary culture, the tumors of mouse allografts were cut into small pieces (approximately 1 mm^3^) in HBSS. After washing two times, the tissues were transferred into a 15-ml tube with 0.25% trypsin of five to six-fold volume and incubated at 37°C for 40 min. 5 ml of DMEM containing 10% FBS was then added to terminate digestion. The cellular suspension was then placed into a new tube and centrifuged at 1000 rpm for 10 min. The cell pellet was resuspended in 5 ml HBSS, and centrifuged at 1000 rpm for 5 min. The cell pellet was then placed into an appropriate volume of mES medium without LIF and the cells were seeded into a 0.1% gelatin coated 60 mm dish at a density of 5×10^5^/ml. Cells were passaged every 3 days and cellular morphology was observed and photographed using Olympus IX81 microscope equipped with a fluorescence device (Olympus, Tokyo, Japan) and analyzed by MetaMorph (Molecular Devices, LLC, Sunnyvale, CA, USA).

Suspension cultures to generate spheroids were performed as described in Dontu *et al* (16). Briefly, single cell of mouse ESC derived CSC or primary culture were plated on 6 cm ultra-low attachment dishes (Corning Incorporated, Corning, NY, USA) with mES medium containing CM without LIF. After they grew, medium was changed to serum-free mESCs medium added Insulin-Transferrin-Selenium-X (ITS-x) (Life Technologies, Grand Island, NY, USA) without LIF. Spheroids cells were recognized after about a week.

To assay tube formation, a 96-well plate was coated with 50 µl/well of Matrigel (Corning Incorporated) by the incubation at 37°C for 30 min. Then the trypsinized mouse ESC derived CSC or primary culture cells were seeded at 5×10^4^ cells/well with 50 µl of EGM-2 medium with growth factors (Lonza, Basel, Switzerland) and cultured for 18 to 24 h.

#### Animal experiments

Healthy 4-week-old C57BL/6J mice were purchased from Charles River Laboratories (Tokyo, Japan). 10^5^ to 10^6^ B6J-LLCcm or B6J-B16cm cells were subcutaneously or intraperitoneally injected into two mice each-before 8 weeks of age. B6J-23^URT^ cells were also injected the same way as a control of these cells. 10^5^ to 10^6^ B6G-LLCcm cells were subcutaneously and intraperitoneally injected into three mice each. B6G-2 cells were also injected the same way as a control. Mice were daily monitored. When size of the tumor became large enough (around 15 mm), mice had been anesthesia with isoflurane using simple inhalation anesthesia machine for small animal experiments (NARCOBIT-E(II); KN-1071; Natsume Seisakusho Co., Ltd, Japan), and flow meter (RK1710; KOFLOC, Japan) and removed the tumor. Mice were sacrificed when tumors were removed.

#### Histologic analysis

Tumors were fixed with 4%-paraformaldehyde in phosphate buffered solution (Nacalai Tesque, Kyoto, Japan) and then processed using a routine wax-embedding procedure for histologic examination. 5-µm-thick sections were stained with hematoxylin and eosin (HE).

#### RNA extraction, cDNA synthesis and quantitative real time PCR

To test the stem cell marker gene expressions in obtained CSCs or primary cultured cells, total RNA was isolated from B6J-LLCcm, B6J-B16cm, and B6G-LLCcm cells with RNeasy Mini Kit (QIAGEN, Hilden, Germany) and then treated with DNase I (Takara Bio, Kusatsu, Japan). 2 µg of RNA was reverse transcribed with SuperScript III First-strand Synthesis System (Invitrogen, Carlsbad, CA, USA). Quantitative real-time PCR was performed with LightCycler 480 SYBR Green I Master mix (Roche, Basel, Switzerland) according to manufacturer's instructions. The sequences of forward and reverse primers used for qPCR were as following: *Sox2*, 5′-TAGAGCTAGACTCCGGGCGATGA-3′ and 5′-TTGCCTTAAACAAGACCACGAAA-3′; *Pou5f1*, 5′-TCTTTCCACCAGGCCCCCGGCTC-3′ and 5′-TGCGGGCGGACATGGGGAGATCC-3′; *Nanog*, 5′-AGGGTCTGCTACTGAGATGCTCTG-3′ and 5′-CAACCACTGGTTTTTCTGCCACCG-3′; *Gapdh*, 5′-AACGGCACAGTCAAGGCCGA-3′ and 5′-ACCCTTTTGGCTCCACCCTT-3′. qPCR was performed by LightCycler 480 Instrument (Roche, Basel, Switzerland) with 400 nM of each primer. Cycling conditions of the used genes were shown in Table I. Gene expression level was normalized with that of *Gapdh* mRNA.

#### Statistical analysis

The results were expressed as the mean ± standard deviation. The statistical significance was assessed by one-way analysis of variance followed by Tukey's post-hoc test for multiple group comparisons. P-value lower than 0.05 was considered as statistically significant.

## Results

### 

#### mESCs cultured in the CM

**Figure 1.**
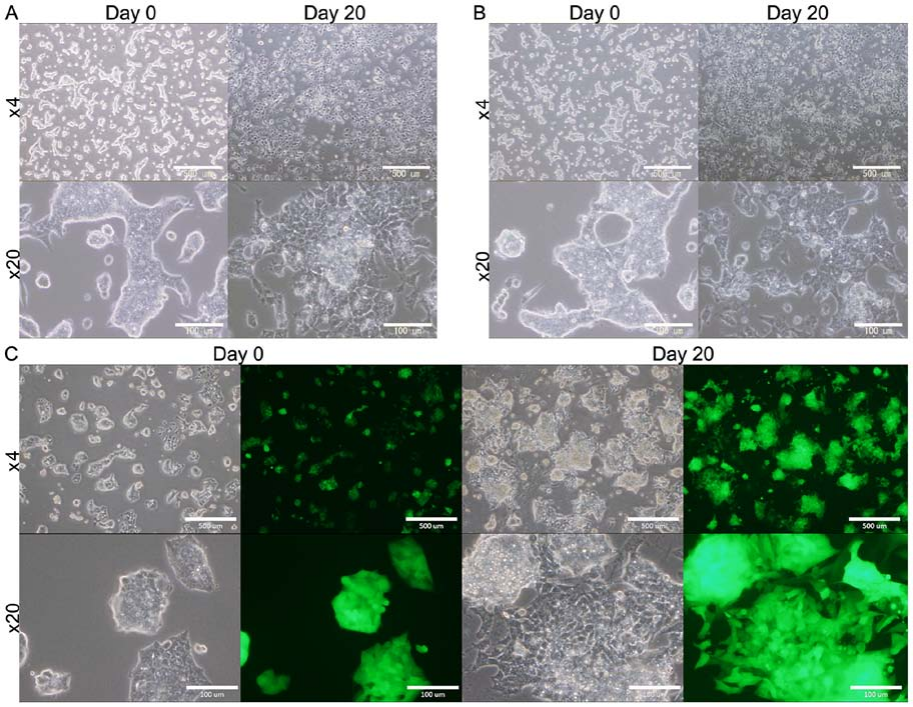
Cell morphology of mouse ESCs at day 0 and 20 during the treatment with the CM. (A) B6J-B16cm cells (B) B6J-LLCcm cells and (C) B6G-LLCcm cells. The morphologies of each ESC appeared changing during the days between Day 0 and Day 20. The morphological change implies the cells differentiated. Scale bars: 500 µm in magnification ×4 and 100 µm in magnification ×20 images. ESCs, embryonic stem cells.

mESCs (B6J-23^URT^, B6G-2 cells) survived for 4 weeks when they were cultured in the presence of CM, while they ceased proliferating and then died in the absence of the CM (Fig. 1). As the controls, mESCs were cultured in mES medium without LIF or in DMEM containing 5% FBS without LIF. However, in both condition the cells gradually differentiated and died during 2 to 3 weeks (data not shown). This expects that the presence of some factors in the CM that provide some survival signals to the cells.

#### Evaluation of tumorigenic ability

**Figure 2.**
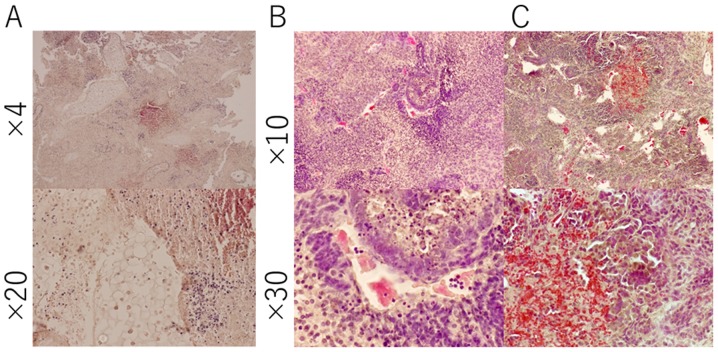
Hematoxylin & eosin staining of the section from the tumor derived from (A) B6J-LLCcm, (B) B6J-B16cm and (C) B6G-LLCcm cells transplanted into mice. These sections were obtained from tumors developed at diaphragm or subcutaneous.

**Figure 3.**
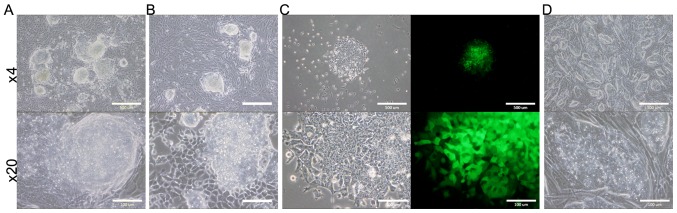
Primary cultures of the tumor cells presented in Fig. 2A. These cells were obtained from (A) B6J-LLCcm, (B) B6J-B16cm, (C) B6G-LLCcm, and (D) the serial transplantation of B6J-LLCcm cells derived from diaphragm or subcutaneous. Scale bars show 500 µm in magnification ×4 and 100 µm in magnification ×20 images.

The survived B6J-LLCcm, B6J-B16cm, and B6G-LLCcm cells, cultured in CM for 30 days, were injected into C57BL/6J mice. The results are summarized in Table II. (Only the total number could be shown, but the both interperitoneally (IP) or subcutaneously (SC) injected mice developed tumor.) The mice transplanted with B6J derived cells developed tumor in about 40 to 60 days and those with B6G derived cells in about 30 days. In each section of the tumors, there were lots of cells infiltrated into the tumor tissue (Fig. 2). Primary cultures of these tumor cells are also shown in Fig. 3. In the primary culture of B6G-LLCcm cells that transplanted in mice, GFP expression was observed (Fig. 3C). This means that the formed tumors are derived from the transplanted cells. When these cultured cells were transplanted into healthy C57BL/6J mice, the tumor appeared again and the stem cell colonies were observed among the primary culture of the tumor cells (Fig. 3D). Moreover, when serial transplantations were done with 5-mm cube of tumors developed from B6J-LLCcm or B6J-B16cm cells, tumors size became larger (data not shown). These results revealed that, the tumors were concluded malignant and they included the population of CSCs.

#### Potential of tube and sphere formation

**Figure 4.**
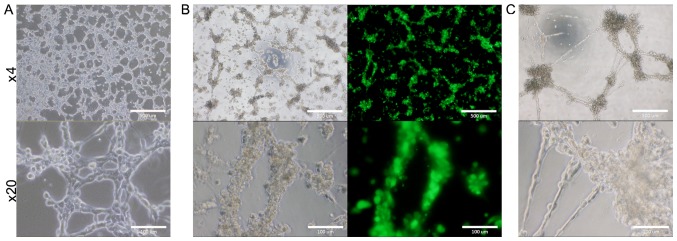
(A) B6J-LLCcm, (B) B6G-LLCcm cells and (C) the primary cultured cells derived from B6J-LLCcm cells formed tube-like luminal structure when they were cultured in EBM-2 medium in the presence of type IV collagen. Each mouse embryonic stem cell treated with the conditioned medium exhibited a differentiation potential. Scale bars: 500 µm in magnification ×4 and 100 µm in magnification ×20 images.

**Figure 5.**
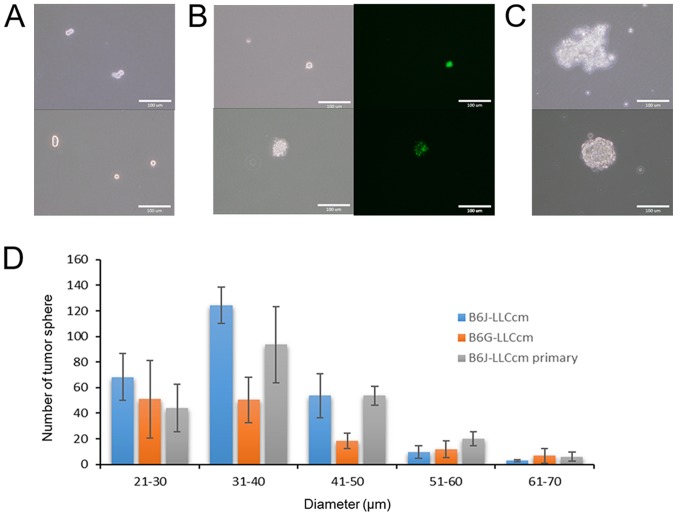
Sphere formation and self-renewal of (A) B6J-LLCcm cells, (B) B6G-LLCcm cells and (C) the primary cultured cells derived from B6J-LLCcm cells. The cells were cultured under non-adherent condition in serum-free medium. Each mouse ESC treated with the CM exhibited a self-renewal potential. Scale bars, 100 µm. (D) Bar graph was represented sphere formation of B6G-LLCcm cells, B6J-LLCcm cells, and the primary cultured cells derived from B6J-LLCcm cells. Spheres were photographed after 14 days culturing in non-adherent culture in serum free mESCs medium supplemented with ITS-x. Images were acquired using an IX81 inverted microscope. The number of spheres was counted and the size was measured using ImageJ software. Results were expressed as the mean ± standard deviation of three independent experiments.

The ability to form tube in the presence of type IV collagen was assessed with B6J-LLCcm and B6G-LLCcm cells. The cells being seeded onto the wells coated with Matrigel, the medium was changed to EGM-2 medium after 24 h. All of cells as well as primary cells from the tumors derived from B6J-LLCcm cells formed vessel-like luminal structures (Fig. 4). Sphere formation was also assessed with B6J-LLCcm and B6G-LLCcm cells. Both cells exhibited spheroids in the non-adherent condition (Fig. 5A and B). The primary cultured cells derived from the tumor of B6J-LLCcm cells also formed sphere structure (Fig. 5C). The size and number of spheres was analyzed; however, no significant differences were observed (Fig. 5D). Our results indicated that, the converted cells exhibited CSC properties of differentiation and self-renewal potentials.

#### Stemness markers expression

**Figure 6.**
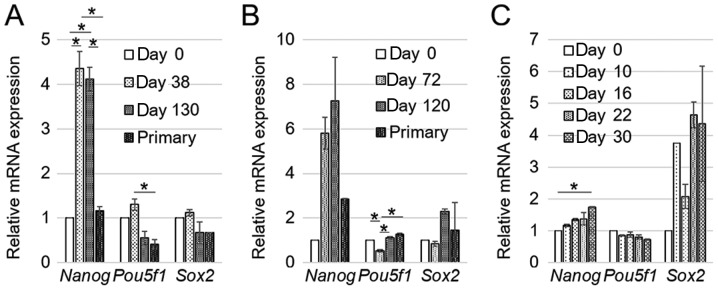
The comparison mRNA expression levels of stem cell markers in mESCs treated with during the treatment with the CM or in the primary cultured cells. The data were expressed as mean ± SD of three independent experiments. (A) B6J-LLCcm (P-values for *Nanog, Pou5f1*, and *Sox2* were 5.63×10^−7^, 0.000104, and 0.000118, respectively), (B) B6J-B16cm (P-values for *Nanog, Pou5f1*, and *Sox2* were 0.0101, 0.000234, 0.169, respectively), and (C) B6G-LLCcm cells (P-values for *Nanog, Pou5f1*, and *Sox2* were 0.00322, 0.0240, and 0.0291, respectively). *P<0.05, as indicated (Tukey's post-hoc test).

To investigate the stemness characteristics of mESCs, the expression of stem cell markers, *Nanog, Pou5f1*, and *Sox2* was assessed by RT-qPCR in B6J-LLCcm, B6J-B16cm, and B6G-LLCcm cells (either at day 0 or after treatment with the CM) as well as in the primary cultured cells of B6J-LLCcm and B6JB16cm. *Nanog* was highly expressed in the cells treated with the CM while the other two genes were expressed as much as in mESCs at day 0. Moreover, the expression levels of the genes in the primary culture cells were similar to those in mESCs at day 0 (Fig. 6A and B). Since *Nanog* is thought to have a key role in maintaining pluripotency (17,18), these results indicate that induced CSCs should keep the potential of differentiation through tumor formation. In B6G-LLCcm cells, the expression of *Nanog* and *Pou5f1* at day 0 was similar. In contrast, the expression of *Sox2* was highly kept during the treatment with the CM (Fig. 6C). This observation may indicate the undifferentiated state of B6G-LLCcm cells as CSCs when the report that high expression of *Sox2* was attributed to poor prognosis in carcinoma (19) is taken into consideration. Meanwhile, the expression of *Nanog* gene in B6G-LLCcm cells is lower than those of B6J-LLCcm and B6J-B16cm cells. This might be the reason of lower rate of oncogenesis in the mice (Table II) as previously found in squamous cell carcinomas (20,21). The expression of those genes might be involved in the progression of cancer but further study is needed.

## Discussion

In the present study, mESCs were successfully demonstrated to be converted into CSCs exhibiting the potential of differentiation and self-renewal together with malignant tumorigenicity followed by a number of infiltrated cells. The GFP expressing cells were found in the developed tumors referring to the converted mESCs is the source cells that formed the tumor. Moreover, CSCs were found expressing the markers of undifferentiated cells implying they kept stemness. As the results, mouse ESCs have been found to be converted into CSCs in a short period when affected by some factors derived from the cancerous microenvironment. In this context, the induction of CSCs should not always depend on gene mutations/translocations. This implies normal stem cells might have a differentiation potential to become a cancer origin. This is not a new concept and have been discussed for a while (22–24). Although most of the people focused on the gene mutation, our results shed light on the initial microenvironment of stem cells. Further investigations are still needed to study the mechanism(s) and factor(s), that responsible for the induction of CSCs. mECSs would be useful for obtaining enough number of CSCs for clarification of the mechanisms that involved in cancer development.

Although mouse and human iPSCs are continuously converted into CSCs (3–6,8,9,25,26), the exact inducing factors are not unknown yet. To find them, microarray gene analysis was performed on these CSCs (25,27) and analysis of CM is also ongoing (data not shown). This induction process might be complicated and could be clarify little by little. Additionally, there are only two cancer cell lines used to obtain CM to induce CSCs. Various kinds of cancer cell lines were already used for the induction but there are still several cancer cell lines. It should be cleared all or not all cell lines are could be used for this. Moreover, both SC and IP injected CSCs are developed tumor in this research. This might be because they are stem-like cells rather than cancer cells. Consequently, tissue-specific cancer could be induced only if they were injected into organ or tissue induction might be needed for the tissue-specific tumor induction.

Immune system is also important for tumor exclusion. Currently, we used C57BL/6J mice with normal immune system is normal, therefore, they don't develop tumors without gene mutation. However, with more than 10^6^ induced CSCs, more than half of them developed malignant tumor. Proven that CSCs were not completely excluded by their immune system and there might be a clue for cancer immune evasion.

## Figures and Tables

**Table I.** Reverse transcription-quantitative PCR conditions of each primer.

Primer	*Gapdh*	Nanog	Pou5f1	*Sox2*
Initial Denaturation	95°C, 10 min	95°C, 10 min	95°C, 10 min	95°C, 10 min
Denaturation	95°C, 10 sec	95°C, 10 sec	95°C, 10 sec	95°C, 10 sec
Annealing	61°C, 10 sec	61°C, 10 sec	58°C, 10 sec	58°C, 10 sec
Extension	72°C, 10 sec	72°C, 15 sec	72°C, 10 sec	72°C, 13 sec
No. of cycles	45	45	45	45

**Table II.** A summary of tumor formation by the transplantation of mouse embryonic stem cells treated with conditioned medium.

Cell type	No. of mice that developed tumors out of total n
B6J-LLCcm	4/4
B6J-B16cm	3/4
B6G-LLCcm	2/6

## Data Availability

All data generated or analyzed during the present study are included in this published article.

## Abbreviations

CSC: cancer stem cell
mESCs: mouse embryonic stem cells
